# Chilean children’s adherence to sustainable healthy diets and its associations with sociodemographic and anthropometric factors: a cross-sectional study

**DOI:** 10.1007/s00394-024-03435-6

**Published:** 2024-06-03

**Authors:** Carolina Venegas Hargous, Liliana Orellana, Camila Corvalan, Claudia Strugnell, Steven Allender, Colin Bell

**Affiliations:** 1 Global Centre for Preventive Health and Nutrition (GLOBE), Institute for Health Transformation, Deakin University, Geelong, Australia; 2 Faculty of Health, School of Medicine, Deakin University, Geelong, Australia; 3 Faculty of Health, Biostatistics Unit, Deakin University, Geelong, Australia; 4 Institute of Nutrition and Food Technology (INTA), University of Chile, Santiago, Chile; 5 Institute for Physical Activity and Nutrition (IPAN), Deakin University, Geelong, Australia

**Keywords:** Sustainable healthy diet, EAT-Lancet diet, Pre-school children, Chilean children

## Abstract

**Purpose:**

To describe adherence to sustainable healthy diets among a sample of 958 Chilean pre-schoolers (3–6 years) and explore associations between adherence and child and maternal sociodemographic and anthropometric characteristics.

**Methods:**

Children’s adherence to sustainable healthy diets was calculated from single multiple-pass 24-h dietary recalls using the Planetary Health Diet Index for children and adolescents (PHDI-C). Higher PHDI-C scores (max score = 150 points) represent greater adherence. Adjusted linear regression models were fitted to explore associations between PHDI-C scores and child and maternal characteristics.

**Results:**

Children obtained low total PHDI-C scores (median 50.0 [IQR 39.5–59.8] points). This resulted from low consumption of *nuts & peanuts*, *legumes*, *vegetables, whole cereals,* and *vegetable oils*; a lack of balance between *dark green* and *red & orange vegetables*, inadequate consumption of *tubers & potatoes* and *eggs & white meats*, and excess consumption of *dairy products*, *palm oil*, *red meats*, and *added sugars*. Mean PHDI-C total score was significantly higher (50.6 [95%CI 49.6, 51.7] vs 47.3 [95%CI 45.0, 49.5]) among children whose mothers were ≥ 25 years compared to those with younger mothers. Positive associations were observed between scores for *fruits* and maternal education, *vegetables* and maternal age, *added sugars* and child weight status, while negative associations were observed between *fruits* and child age, and *vegetable oils* and maternal education. Scores for *dairy products* PHDI-C component were lower among girls.

**Conclusion:**

Adherence to sustainable healthy diets was low among this sample of Chilean children and was significantly associated with maternal age, being lower among children whose mothers were younger.

**Supplementary Information:**

The online version contains supplementary material available at 10.1007/s00394-024-03435-6.

## Introduction

Adopting healthy and environmentally sustainable diets (hereafter sustainable healthy diets) early in life is key to preventing multiple forms of malnutrition and mitigating climate change [1, 2]. Such diets are meant to reduce the risk of diet-related non-communicable diseases and food-borne diseases, be nutritionally adequate, have a low environmental impact, be affordable, equitable, and culturally acceptable for all [3]. Ideally, they should begin with exclusive breastfeeding for the first six months of life and continue with breastfeeding and complementary feeding until two years of age [3]. Once established in childhood, healthy dietary patterns are likely to be sustained throughout life [4] as long as people have access to diets that are rich in a wide variety of minimally-processed plant-based foods, low in ultra-processed foods that contain excess amounts of saturated fats, added sugars, and/or salt, and moderate in animal-based foods [3].

In 2019, the EAT-Lancet Commission proposed a reference diet for the population aged 2 years and older which can reduce the global greenhouse gas emissions by up to 80% [1]. If universally adopted, it has the potential to ensure sufficient food for 10 billion people in 2050, while staying within planetary boundaries for food production [1]. Chile, Brazil, and Argentina are among the countries that would benefit the most from shifting to sustainable healthy diets, where the per capita carbon footprint could be reduced by up to 75% [5]. However, the cost of the EAT-Lancet diet was estimated to be unaffordable for 1.58 billion people in the world [6], with the highest costs reported for Latin America and the Caribbean [6], suggesting that poorer nations might be less able to adhere to sustainable healthy diets due to an economic disadvantage.

Studies of diets in Latin America show poor alignment with the EAT-Lancet diet [7–10]; however, only a few included children in the analysis [7, 8]. There is only one study internationally that has provided a detailed description of children’s adherence to sustainable healthy diets [11]. This study, conducted by Bäck et al. [11], indicated that Finnish pre-schoolers’ diets were far from meeting the EAT-Lancet dietary targets due to low consumption of nuts, legumes, whole grains, vegetables, and unsaturated oils, and high consumption of red meat, dairy products, tubers and potatoes, and added sugars [11]. The absence of studies in children is a glaring gap considering they comprise almost 30% of the world’s population [12]. Further, a systematic review on predictors of children’s dietary intake found that maternal education and socio-economic position may be key determining factors [13], and a recent scoping review found that children’s diet quality was associated with their weight status [14]. This suggests that adherence to sustainable healthy diets might also be determined by child and maternal socio-demographic and anthropometric characteristics. However, no empirical evidence currently exists.

To help address the lack of studies in children and limited knowledge of determinants of sustainable healthy diets, we aimed to describe adherence to sustainable healthy diets among a sample of Chilean pre-schoolers and explore associations between adherence and child and maternal sociodemographic and anthropometric characteristics.

## Subjects and methods

### Study design and participants

This study is a secondary analysis of cross-sectional data collected in 2016 from the Food Environment Chilean Cohort (FECHiC). The original study design and recruitment process have been described in detail elsewhere [15]. Briefly, 961 children aged 3–6-years-old were recruited from public schools located in low-medium income neighbourhoods of south-eastern Santiago, Chile. Participants are a convenience sample, but they have similar socioeconomic and anthropometric characteristics to children living in low-medium income neighbourhoods of urban areas across the country [16, 17].

### Data collection

Baseline data collection for FECHiC was conducted between April to August of 2016. Trained dietitians obtained sociodemographic data (i.e., children’s age, gender, maternal age, and level of maternal education) from children’s primary caretakers (usually mothers) and measured child and maternal anthropometric characteristics (weight, height, waist circumference) following standardized procedures [18]. Children’s dietary intake data was obtained from their primary caretakers using single 24-h recalls following the US Department of Agriculture (USDA) automated multiple pass method [19], which helps reduce the risk of recall bias [20]. A photographic atlas of Chilean foods and culinary preparations [21] was used to aid estimation of portion sizes. Dietary recall characteristics such as day of the recall (weekday vs weekend/holiday), type of eating pattern on the day of recall (typical vs atypical (because of celebration, or sickness, or vacation)), type of diet on the day of recall (normal (i.e., omnivorous with no dietary restriction of any kind) vs special (i.e., lactose free, gluten free, vegetarian, or vegan)), and reliability of the recall (reliable (i.e., recalls with no missing information) vs unreliable (i.e., recalls with missing information on the amount consumed of some food items) were also collected. Three participants with unavailable dietary data were excluded from this study. The final sample included 958 participants (Supplemental Fig. 1).

### Linkage of dietary data with nutrient composition and ingredients list data

Dietary intake data was linked to a bespoke food composition database developed for Chile by the University of North Carolina and the Institute of Nutrition and Food Technology (INTA) that included data from the USDA National Nutrient Database [22] and from nutrition information panels and ingredients list of packaged products available in Chile during the first quarter of 2016 [23]. Once the linkage process was completed, children’s energy and nutritional intake were determined.

### Outcomes of interest

Children’s adherence to sustainable healthy diets was quantified using the Planetary Health Diet Index for children and adolescents (PHDI-C) [24]; an adaptation of the Planetary Health Diet Index (PHDI) developed and validated by Cacau et al. [25]. The PHDI-C follows the EAT-Lancet Commission’s dietary recommendations [1] with five modifications to better reflect children’s and adolescents’ micronutrient requirements [24]. It comprises 16 components: four adequacy components for food groups that children are encouraged to eat more of (i.e., *nuts & peanuts*, *legumes*, *fruits*, and *vegetables*); three ratio components to promote consumption of a variety of vegetables and cereals (i.e., *dark green vegetables ratio*, *red and orange vegetables ratio*, and *whole cereals ratio*); five optimum components for foods whose consumption should be balanced to achieve diet quality and environmental sustainability (i.e., *cereals, tubers & potatoes, dairy products, eggs & white meats,* and *vegetable oils*); and four moderation components for foods that children should eat less of (i.e., *palm oil, red meats, animal fats,* and *added sugars*) [24]. Each component is associated with a range of recommended percentages of total energy intake and a continuous scoring scale. Components can score between 0 to 10 points, except for the *dark green vegetables* and *red and orange vegetables* ratio components where the maximum score is 5 points. The formulae to calculate each PHDI-C component score are provided in detail elsewhere [24]. For adequacy components, consumption equal to or above the recommended percentage of total energy intake is given the highest score while consumption below the recommended percentage of total energy intake is given proportionally lower scores; for ratio and optimum components, consumption equal to or close to the recommended percentage of total energy intake is given higher scores while consumption either above or below the recommended percentage of total energy intake is given proportionally lower scores; lastly, for moderation components, consumption equal to or close to zero is given higher scores while consumption above zero is given proportionally lower scores. Of note is that consumptions outside the recommended range of percentage of total energy intake for each component are given zero points. PHDI-C component scores are then added, resulting in a total score ranging from 0 to 150 points (see Table 2, columns 1–3) [24]. Higher total scores indicate higher adherence to sustainable healthy diets.

### Independent variables

We explored associations between children’s PHDI-C total and individual component scores and child and maternal characteristics, including child gender (female or male), child age (3–4 years or 5–6 years), maternal age (categorized as < 25 years or ≥ 25 years to distinguish between young mothers and older mothers, respectively), and maternal education (incomplete secondary education, complete secondary education, or complete tertiary education), which was used as a proxy of socioeconomic position [26]. Weight and height were used to calculate child and maternal body mass index (BMI) and determine weight status according to appropriate WHO Child Growth Standards [27, 28] and adults cut-points [29]. A small proportion of children (2.4%) and mothers (0.5%) were at risk of undernutrition, and they were classified as non-overweight along with those in the healthy weight range. Maternal waist circumference was used to determine the presence of abdominal obesity on children’s mothers (waist circumference > 88 cm) [30].

### Statistical analysis

Total PHDI-C scores and individual component scores were reported using descriptive statistics. Additionally, we reported the proportion of participants whose scores were (1) < 25% of the maximum score; (2) ≥ 25% and < 50%; (3) ≥ 50% and < 75%; and ≥ 75% of the maximum score.

Linear regression models were fitted to explore whether children’s PHDI-C total and individual component scores (dependent variables) were associated with child and maternal sociodemographic and anthropometric characteristics. All models included all maternal and child characteristics plus three dietary recall characteristics that were significantly associated with PHDI-C total scores: day of dietary recall (weekday vs weekend/holiday); type of eating pattern on the day of the recall (typical vs atypical); and type of diet on the day of the recall (normal vs special) [24]. We reported adjusted estimates alongside 95% confidence intervals (CI). Only participants with complete dietary, sociodemographic, and anthropometric data were included in this analysis (n = 917) (Supplementary Fig. 1). Forty-one children were excluded due to missing anthropometric data (one child and two mothers refused to be measured, and 38 mothers were not measured due to pregnancy). Statistical analysis was conducted in Stata v17. Statistical significance was defined as p-value < 0.05.

## Results

### Participants socio-demographic, anthropometric and dietary recall characteristics (Table 1)

**Table 1:** Sociodemographic, anthropometric, and dietary recall characteristics of FECHiC participants (n = 958)

	n	(%)
**Sociodemographic characteristics**		
Child gender		
Male	462	(48.2)
Female	496	(51.8)
Child age		
3–4 years	695	(72.6)
5–6 years	263	(27.4)
Maternal age		
< 25 years	167	(17.4)
≥ 25 years	791	(82.6)
Maternal level of education		
Incomplete secondary education	173	(18.1)
Complete secondary education	529	(55.2)
Complete tertiary education	256	(26.7)
**Anthropometric characteristics**		
Child weight status^a,b^		
At risk of undernutrition	23	(2.4)
Normal weight	483	(50.5)
Overweight	275	(28.7)
Obesity	118	(12.3)
Severe obesity	58	(6.1)
Maternal weight status^c,d^		
Underweight	5	(0.5)
Normal weight	241	(26.3)
Overweight	345	(37.6)
Obesity class I	217	(23.6)
Obesity class II	75	(8.2)
Obesity class III	35	(3.8)
Maternal abdominal obesity^d,e^		
Absence	408	(44.4)
Presence	510	(55.6)
**Dietary recall characteristics**		
Day of the dietary recall		
Weekday	821	(85.7)
Weekend day/holiday	137	(14.3)
Type of eating pattern on the day of the dietary recall^f^		
Typical	801	(83.6)
Atypical	157	(16.4)
Type of diet on the day of the dietary recall^g^		
Normal	905	(94.5)
Special	53	(5.5)
Reliability of the dietary recall^h^		
Reliable	904	(94.4)
Unreliable	54	(5.6)

Abbreviation: *FECHiC* Food Environment Chilean Cohort

^a^Child weight status was defined according to WHO Child Growth Standards 2006 [27] for children under 5-years-old, and WHO Growth Reference 2007 [28] for children above 5-years of age

^b^Missing data for one child who refused to be measured

^c^Maternal weight status was defined using the WHO cut-off points for BMI in adults [49]

^d^Missing data for 40 participants; 38 mothers were pregnant and two refused to be measured

^e^Presence of abdominal obesity in children’s mothers was defined using the Adult Treatment Panel III criteria for clinical identification of metabolic syndrome (waist circumference above 88 cm) [30]

^f^Typical eating pattern refers to a recall from a regular day; atypical eating pattern refers to a recall from a special occasion such celebration, vacation, or sickness

^g^Normal diet refers to an omnivorous diet with no dietary restriction of any kind; special diet refers to lactose free, gluten free, vegetarian, or vegan diets

^h^Unreliable recalls refer to recalls where there was missing information on the amount consumed of some food items

In 2016, most children (72.6%) were 3–4 years and 51.8% were female. Their mothers were mostly ≥ 25 years (82.6%), with 55.2% having completed secondary and 26.7% having completed tertiary education. Half of children had normal weight status, 28.7% were living with overweight, and 18.4% were living with obesity. Most mothers (70.2%) were living with either overweight or obesity, and 53.2% presented abdominal obesity. Dietary recalls were mostly from weekdays (85.7%) and were reported by primary caretakers as children’s typical eating pattern (83.6%). Most participants (94.5%) reported having a normal diet on the day of the dietary recall. Six percent of recalls were classified as unreliable by INTA’s dietetic team because of missing information on some food items.

### Children’s PHDI-C scores (Table 2)

Adherence to sustainable healthy diets was low (median PHDI-C total score = 50.0 points), with the majority (95.4%, n = 914) of children obtaining < 75 points.

**Table 2:** PHDI-C components with recommended percentages of total energy intakes and possible scores along with FECHiC participants’ median percentage of total energy intake by PHDI-C component, corresponding scores, and proportion of participants below or above maximum scores (n = 958)

PHDI-C components	PHDI-C recommended percentage of total energy intake for children^a^	PHDI-C possible scores	Participants’ percentage of total energy intake^a^	Participants’ PHDI-C scores^b^	Participants < 25% of max score	Participants ≥ 25% & < 50% of max score	Participants ≥ 50% & < 75% of max score	Participants ≥ 75% of max score
	% kcal (range)	Points	Median % kcal (IQR)	Median score (IQR)	n (%)	n (%)	n (%)	n (%)
Adequacy components								
Nuts & peanuts	≥ 11.6 (0.0–100)	0–10	0.0 (0.0–0.0)	0.0 (0.0–0.0)	933 (97.4)	9 (0.9)	7 (0.7)	9 (0.9)
Legumes	≥ 11.3 (0.0–100)	0–10	0.0 (0.0–0.7)	0.0 (0.0–0.6)	797 (83.2)	19 (2.0)	29 (3.0)	113 (11.8)
Fruits	≥ 5.0 (0.0–100)	0–10	3.6 (0.4–7.7)	7.2 (0.8–10.0)	286 (29.8)	89 (9.3)	112 (11.7)	471 (49.2)
Vegetables	≥ 3.1 (0.0–100)	0–10	1.1 (0.5–2.0)	3.6 (1.6–6.4)	346 (36.1)	279 (29.1)	152 (15.9)	181 (18.9)
**Ratio components**								
DGV ratio	29.5 (0.0–100)	0–5	0.0 (0.0–0.0)	0.0 (0.0–0.0)	855 (89.3)	31 (3.2)	39 (4.1)	33 (3.4)
ReV ratio	38.5 (0.0–100)	0–5	41.0 (11.2–59.5)	2.7 (0.0–4.0)	316 (33.0)	140 (14.6)	229 (23.9)	273 (28.5)
WC ratio	75.0 (0.0–100)	0–10	0.0 (0.0–0.9)	0.0 (0.0–0.1)	876 (91.4)	52 (5.4)	20 (2.1)	10 (1.0)
**Optimum components**								
Cereals	30.0 (0.0–60.0)	0–10	24.6 (18.5–31.0)	7.5 (5.7–8.8)	35 (3.7)	149 (15.5)	298 (31.1)	476 (49.7)
Tubers & potatoes	1.6 (0.0–3.1)	0–10	0.0 (0.0–5.8)	0.0 (0.0–0.0)	886 (92.5)	26 (2.7)	23 (2.4)	23 (2.4)
Dairy products	12.2 (0.0–24.4)	0–10	19.1 (12.6–26.0)	3.6 (0.0–7.0)	423 (44.1)	148 (15.5)	190 (19.8)	197 (20.6)
Eggs & white meats	6.2 (0.0–12.2)	0–10	3.8 (0.0–8.9)	0.7 (0.0–5.8)	552 (57.6)	130 (13.6)	132 (13.8)	144 (15.0)
Vegetable oils	14.1 (0.0–28.3)	0–10	9.6 (5.5–14.2)	5.8 (3.5–7.9)	160 (16.7)	220 (23.0)	290 (30.3)	288 (30.0)
**Moderation components**								
Palm oil	0.0 (0.0–2.4)	0–10	1.3 (0.0–5.6)	4.4 (0.0–10.0)	443 (46.2)	47 (4.9)	91 (9.5)	377 (39.4)
Red meats	0.0 (0.0–2.4)	0–10	1.6 (0.0–6.7)	3.2 (0.0–10.0)	474 (49.5)	21 (2.2)	7 (0.7)	456 (47.6)
Animal fats	0.0 (0.0–1.4)	0–10	0.0 (0.0–2.7)	10.0 (0.0–10.0)	415 (43.3)	35 (3.6)	15 (1.6)	493 (51.5)
Added sugars	0.0 (0.0–4.8)	0–10	15.3 (11.1–21.2)	0.0 (0.0–0.0)	926 (96.7)	21 (2.2)	4 (0.4)	7 (0.7)
Total	–	0–150	–	50.0 (39.5–59.8)	191 (19.9)	723 (75.5)	44 (4.6)	0 (0.0)

Abbreviations: *FECHiC* Food Environment Chilean Cohort, *PHDI-C* Planetary Health Diet Index for children and adolescents, *DGV* ratio, dark green vegetables ratio, *ReV ratio* red and orange vegetables ratio, *WC ratio* whole cereals ratio, *% kcal* percentage of energy intake, *IQR* interquartile range; max, maximum

^a^Values are expressed as percentage of total energy intake, except for the DGV ratio & ReV ratio components where values are expressed as percentage of total energy intake from vegetables, and for the WC ratio component where values are expressed as percentage of total energy intake from cereals

^b^The formulae to calculate each PHDI-C component score are provided in detail elsewhere [24]. For adequacy components, consumption equal to or above the recommended percentage of total energy intake is given the highest score while consumption below the recommended percentage of total energy intake is given proportionally lower scores; for ratio and optimum components, consumption equal to or close to the recommended percentage of total energy intake is given higher scores while consumption either above or below the recommended percentage of total energy intake is given proportionally lower scores; lastly, for moderation components, consumption equal to or close to zero is given higher scores while consumption above zero is given proportionally lower scores. Of note is that consumptions outside the recommended range of percentage of total energy intake for each component are given zero points

For adequacy components, median percentage of total energy intake from *nuts & peanuts*, *legumes*, and *vegetables* were below recommended intakes. Consequently, 97.4%, 83.2% and 36.1% of children obtained < 2.5 points for each component, respectively. In contrast, median percentage of total energy intake from *fruits* was closer to the recommended intake, with 49.2% (n = 471) obtaining ≥ 7.5 points.

For ratio components, median percentage of total energy intake from *dark green vegetables* and *whole cereals* were negligible, with ~ 90% of participants (n = 855 and n = 876, respectively) obtaining < 1.25 points. Median percentage of total energy intake from *red & orange vegetables* was slightly higher than recommended, with 28.5% of children (n = 273) obtaining ≥ 3.75 points.

For optimum components, median percentage of total energy intake from *cereals* was close to the recommended intake, resulting in 50% (n = 476) of children obtaining ≥ 7.5 points. Consumption of *tuber & potatoes* was either negligible for at least 50% of participants or excessive for at least 25% of participants, meaning the majority (93%, n = 886) obtained < 2.5 points. Consumption of *dairy products* was higher than recommended for at least 75% of participants, resulting in 44.1% of participants (n = 423) obtaining < 2.5 points. Consumption of *eggs & white meats* was lower than recommended for at least 50% of participants and higher than recommended for at least 25% of participants, meaning that almost 60% of participants (n = 552) obtained < 2.5 points. Consumption of *vegetable oils* was lower than recommended for most children, with only 30.0% (n = 288) obtaining ≥ 7.5 points.

Finally, median percentage of total energy intake from moderation components *palm oil, red meat,* and *added sugars* were higher than recommended, with 46.2%, 49.5%, and 96.7% of participants obtaining < 2.5 points, respectively. Consumption of *animal fats* was close to the recommended intake for at least 50% of participants, meaning 51.5% of participants obtained ≥ 7.5 points.

### Associations between PHDI-C total score and sociodemographic and anthropometric characteristics (Table 3)

**Table 3:** Adjusted associations between FECHiC participants’ PHDI-C total score and sociodemographic and anthropometric characteristics

	PHDI-C total score^a,b,e^				
	Mean	95% CI	Diff	95% CI	P-value
Total sample	50.04	(49.10, 50.97)			
**Sociodemographic characteristics**					
Child gender					
Male	50.51	(49.15, 51.86)			
Female	49.60	(48.31, 50.90)	−0.90	(−2.78, 0.97)	0.345
Child age					
3–4 years	50.46	(49.36, 51.57)			
5–6 years	48.92	(47.12 50.72)	−1.54	(−3.67, 0.59)	0.156
Maternal age					
< 25 years	47.26	(44.97, 49.54)			
≥ 25 years	50.63	(49.60, 51.66)	3.37	(0.85, 5.90)	0.009*
Maternal level of education					
Incomplete secondary education	49.73	(47.52, 51.94)			
Complete secondary education	49.95	(48.69, 51.21)	0.22	(−2.32, 2.76)	0.863
Complete tertiary education	50.41	(48.58, 52.25)	0.68	(−2.23, 3.59)	0.645
**Anthropometric characteristics**					
Child weight status^c^					
Non overweight	49.85	(48.57, 51.14)			
Overweight	49.75	(47.99, 51.51)	−0.10	(−2.29, 2.09)	0.926
Obesity	51.02	(48.78, 53.27)	1.17	(−1.44, 3.78)	0.379
Maternal weight status^d^					
Non overweight	50.79	(48.47, 53.13)			
Overweight	49.56	(48.03, 51.09)	−1.23	(−3.97, 1.50)	0.376
Obesity	49.96	(47.98, 51.95)	−0.83	(−4.43, 2.77)	0.650
Maternal abdominal obesity^d^					
Absence	49.25	(47.43, 51.06)			
Presence	50.67	(49.11, 52.23)	1.42	(−1.38, 4.23)	0.319

Abbreviations: *PHDI-C* Planetary Health Diet Index for children and adolescents, *FECHiC* Food Environment Chilean Cohort, *CI* confidence interval, *diff* difference (i.e., regression coefficient)

^a^PHDI-C total score range: 0–150 points

^b^Estimates and p-values from linear regression models adjusting for all characteristics listed in the table plus dietary recall characteristics including: day of the dietary recall (weekday vs weekend/holiday), type of eating pattern (typical (i.e., recall from a typical day) vs atypical (i.e., recall from a special occasion such as celebrations, sickness or vacations)), and type of diet (normal (i.e., omnivorous diet with no dietary restriction of any kind) vs special diet (e.g., lactose free, gluten free, vegan or vegetarian diets)); n = 917

^c^Missing data for 1 child who refused to be measured

^d^Missing data for 40 mothers; 38 were pregnant and two refused to be measured

^e^Adjusted R-squred = 0.0184

*P-value < 0.05

Mean total PHDI-C score was significantly higher for participants whose mothers were ≥ 25 years old compared to those whose mothers were younger (50.6 vs 47.3 points, p-value 0.009). No other statistically significant associations were observed.

### Associations between individual component scores and sociodemographic and anthropometric characteristics (Table 4)

Among adequacy components, mean score for *fruits* was significantly lower for children aged 5–6 years compared to children aged 3–4 years (5.3 vs 6.2 points, p-value 0.004), and significantly higher for children’s whose mothers had completed tertiary education compared to those whose mothers had not completed secondary education (6.4 vs 5.5 points, p-value 0.038). Mean score for *vegetables* was significantly higher for children whose mothers were ≥ 25 years compared to those with younger mothers (4.2 vs 3.6 points, p-value 0.025). We did not observe significant associations between ratio component scores and sociodemographic or anthropometric characteristics. Among optimum components, mean score for *dairy products* was significantly lower for females compared to males (3.5 vs 4.0 points, p-value 0.023). Mean score for *vegetable oils* was significantly lower for children whose mothers had completed secondary or tertiary education, compared to those whose mothers had not completed secondary education (5.5 and 5.4 vs 6.0 points, respectively; p-value 0.032 and 0.023, respectively). Finally, among moderation components, mean score for *added sugars* was significantly higher for children living with obesity compared to children not living with overweight/obesity (0.3 vs 0.1 points, p-value 0.037).

**Table 4:** Adjusted associations between FECHiC participants’ PHDI-C component scores and sociodemographic and anthropometric characteristics

	Nuts & peanuts comp. scores^a,b,e^			Legumes component scores^a,b,f^			Fruits component scores^a,b,g^			Vegetables component scores^a,b,h^		
	Mean (95% CI)	Diff (95% CI)	P-value	Mean (95% CI)	Diff (95% CI)	P-value	Mean (95% CI)	Diff (95% CI)	P-value	Mean (95% CI)	Diff (95% CI)	P-value
**Sociodemographic characteristics**												
Child gender												
Male	0.16 (0.05, 0.27)			1.66 (1.36, 1.96)			6.07 (5.68, 6.45)			3.94 (3.64, 4.23)		
Female	0.25 (0.15, 0.36)	0.09 (−0.06, 0.24)	0.238	1.49 (1.20, 1.77)	−0.17 (−0.59, 0.25)	0.424	5.84 (5.47, 6.21)	−0.23 (−0.77, 0.31)	0.408	4.21 (3.93, 4.49)	0.27 (−0.14, 0.68)	0.190
Child age												
3–4 years	0.19 (0.10, 0.28)			1.65 (1.40, 1.90)			6.20 (5.88, 6.51)			4.14 (3.90, 4.38)		
5–6 years	0.28 (0.13, 0.42)	0.09 (−0.08, 0.26)	0.292	1.35 (0.95, 1.75)	−0.30 (−0.77, 0.18)	0.222	5.30 (4.78, 5.81)	−0.90 (−1.51, −0.29)	0.004*	3.93 (3.54, 4.32)	−0.21 (−0.67, 0.26)	0.380
Maternal age												
< 25 years	0.12 (−0.07, 0.30)			1.44 (0.94, 1.95)			5.51 (4.86, 6.17)			3.56 (3.06, 4.06)		
≥ 25 years	0.23 (0.15, 0.32)	0.12 (−0.09, 0.32)	0.267	1.59 (1.36, 1.82)	0.15 (−0.41, 0.71)	0.598	6.04 (5.74, 6.34)	0.52 (−0.20, 1.25)	0.156	4.19 (3.97, 4.42)	0.63 (0.08, 1.18)	0.025*
Maternal level of education												
Incomplete SE	0.18 (−0.00, 0.36)			1.50 (1.01, 1.99)			5.51 (4.88, 6.15)			3.84 (3.36, 4.32)		
Complete SE	0.19 (0.09, 0.30)	0.01 (−0.19, 0.22)	0.889	1.46 (1.18, 1.74)	−0.03 (−0.60, 0.53)	0.905	5.87 (5.51, 6.23)	0.36 (−0.37, 1.09)	0.337	4.09 (3.82, 4.37)	0.26 (−0.30, 0.81)	0.366
Complete TE	0.27 (0.12, 0.42)	0.09 (−0.15, 0.32)	0.466	1.83 (1.42, 2.24)	0.33 (−0.32, 0.98)	0.317	6.40 (5.87, 6.93)	0.89 (0.05, 1.72)	0.038*	4.21 (3.81, 4.61)	0.37 (−0.26, 1.01)	0.249
**Anthropometric characteristics**												
Child weight status^c^												
Non overweight	0.25 (0.14, 0.35)			1.63 (1.34, 1.91)			5.75 (5.38, 6.12)			4.08 (3.79, 4.36)		
Overweight	0.20 (0.05, 0.34)	−0.05 (−0.23, 0.13)	0.582	1.78 (1.39, 2.17)	0.15 (−0.34, 0.64)	0.546	6.07 (5.57, 6.58)	0.33 (−0.30, 0.96)	0.306	4.11 (3.72, 4.49)	0.03 (−0.45, 0.51)	0.903
Obesity	0.14 (−0.05, 0.32)	−0.11 (−0.32, 0.10)	0.306	1.05 (0.55, 1.55)	−0.57 (−1.15, 0.01)	0.053	6.34 (5.70, 6.98)	0.59 (−0.16, 1.34)	0.122	4.05 (3.56, 4.54)	−0.03 (−0.59, 0.54)	0.929
Maternal weight status^d^												
Non overweight	0.07 (−0.11, 0.26)			1.84 (1.32, 2.36)			5.97 (5.30, 6.64)			4.16 (3.66, 4.67)		
Overweight	0.18 (0.06, 0.30)	0.11 (−0.12, 0.33)	0.349	1.87 (1.53, 2.21)	0.03 (−0.58, 0.64)	0.932	5.80 (5.36, 6.24)	−0.17 (−0.95, 0.62)	0.679	3.79 (3.46, 4.13)	−0.37 (−0.96, 0.23)	0.225
Obesity	0.35 (0.19, 0.51)	0.27 (−0.02, 0.57)	0.065	1.04 (0.60, 1.49)	−0.80 (−1.60,−0.00)	0.051	6.09 (5.52, 6.66)	0.12 (−0.92, 1.15)	0.825	4.32 (3.89, 4.75)	0.15 (−0.63, 0.94)	0.700
Maternal abdominal obesity^d^												
Absence	0.28 (0.13, 0.43)			1.27 (0.86, 1.67)			5.86 (5.34, 6.38)			4.24 (3.84, 4.64)		
Presence	0.16 (0.03, 0.28)	−0.12 (−0.35, 0.10)	0.286	1.81 (1.46, 2.15)	0.54 (−0.09, 1.16)	0.091	6.02 (5.57, 6.46)	0.15 (−0.65, 0.96)	0.707	3.95 (3.61, 4.29)	−0.29 (−0.90, 0.32)	0.352

	DGV ratio component scores^a,b,i^			ReV ratio component scores^a,b,j^			WC ratio component scores^a,b,k^			Cereals component scores^a,b,l^		
	Mean (95% CI)	Diff (95% CI)	P-value	Mean (95% CI)	Diff (95% CI)	P-value	Mean (95% CI)	Diff (95% CI)	P-value	Mean (95% CI)	Diff (95% CI)	P-value
**Sociodemographic characteristics**												
Child gender												
Male	0.39 (0.29, 0.48)			2.44 (2.27, 2.61)			0.51 (0.37, 0.65)			7.06 (6.85, 7.26)		
Female	0.32 (0.23, 0.42)	−0.06 (−0.20, 0.07)	0.352	2.33 (2.18, 2.50)	−0.11 (−0.34, 0.13)	0.376	0.61 (0.48, 0.74)	0.10 (−0.09, 0.29)	0.311	7.01 (6.81, 7.20)	−0.05 (−0.34, 0.24)	0.732
Child age												
3–4 years	0.39 (0.31, 0.47)			2.46 (2.32, 2.60)			0.53 (0.42, 0.64)			7.03 (6.86, 7.19)		
5–6 years	0.26 (0.13, 0.39)	−0.13 (−0.28, 0.03)	0.108	2.21 (1.98, 2.43)	−0.25 (−0.51, 0.02)	0.068	0.63 (0.45, 0.82)	0.10 (−0.11, 0.32)	0.350	7.03 (6.76, 7.31)	−0.00 (−0.32, 0.33)	0.987
Maternal age												
< 25 years	0.22 (0.05, 0.38)			2.20 (1.92, 2.49)			0.35 (0.12, 0.58)			7.22 (6.87, 7.57)		
≥ 25 years	0.38 (0.31, 0.46)	0.16 (−0.02, 0.35)	0.077	2.43 (2.30, 2.56)	0.23 (−0.09, 0.54)	0.162	0.60 (0.50, 0.71)	0.25 (−0.00, 0.51)	0.054	6.99 (6.83, 7.15)	−0.23 (−0.61, 0.16)	0.245
Maternal level of education												
Incomplete SE	0.31 (0.15, 0.47)			2.51 (2.23, 2.78)			0.49 (0.26, 0.71)			7.08 (6.75, 7.42)		
Complete SE	0.37 (0.28, 0.47)	0.06 (−0.12, 0.24)	0.519	2.44 (2.28, 2.59)	−0.07 (−0.39, 0.25)	0.666	0.52 (0.40, 0.65)	0.04 (−0.22, 0.29)	0.781	7.13 (6.94, 7.33)	0.05 (−0.34, 0.44)	0.801
Complete TE	0.33 (0.20, 0.47)	0.02 (−0.19, 0.23)	0.849	2.21 (1.98, 2.44)	−0.30 (−0.66, 0.07)	0.109	0.68 (0.50, 0.87)	0.20 (−0.10, 0.49)	0.186	6.78 (6.50, 7.06)	−0.30 (−0.75, 0.14)	0.183
**Anthropometric characteristics**												
Child weight status^c^												
Non overweight	0.35 (0.26, 0.44)			2.37 (2.21, 2.53)			0.63 (0.51, 0.77)			7.06 (6.86, 7.25)		
Overweight	0.34 (0.21, 0.46)	−0.01 (−0.17, 0.15)	0.875	2.39 (2.17, 2.61)	0.02 (−0.25, 0.29)	0.880	0.42 (0.24, 0.60)	−0.21 (−0.44, 0.01)	0.058	6.90 (6.63, 7.17)	−0.16 (−0.49, 0.18)	0.352
Obesity	0.39 (0.22, 0.55)	0.04 (−0.15, 0.22)	0.711	2.46 (2.17, 2.74)	0.09 (−0.24, 0.42)	0.595	0.55 (0.33, 0.78)	−0.08 (−0.35, 0.18)	0.539	7.16 (6.82, 7.51)	0.11 (−0.29, 0.51)	0.597
Maternal weight status^d^												
Non overweight	0.27 (0.10, 0.44)			2.46 (2.16, 2.75)			0.58 (0.34, 0.81)			7.19 (6.84, 7.55)		
Overweight	0.44 (0.33, 0.55)	0.17 (−0.03, 0.37)	0.094	2.29 (2.09, 2.47)	−0.17 (−0.51, 0.17)	0.329	0.62 (0.46, 0.77)	0.04 (−0.24, 0.32)	0.774	6.95 (6.71, 7.18)	−0.25 (−0.67, 0.17)	0.243
Obesity	0.32 (0.18, 0.46)	0.05 (−0.21, 0.31)	0.720	2.45 (2.20, 2.69)	−0.01 (−0.46, 0.44)	0.965	0.49 (0.28, 0.69)	−0.09 (−0.46, 0.27)	0.619	6.99 (6.69, 7.30)	−0.20 (−0.75, 0.35)	0.473
Maternal abdominal obesity^d^												
Absence	0.40 (0.27, 0.53)			2.41 (2.18, 2.64)			0.56 (0.38, 0.75)			6.94 (6.66, 7.21)		
Presence	0.31 (0.20, 0.43)	−0.09 (−0.29, 0.11)	0.394	2.37 (2.18, 2.57)	−0.04 (−0.39, 0.31)	0.833	0.56 (0.40, 0.72)	0.00 (−0.29, 0.28)	0.980	7.11 (6.87, 7.34)	0.17 (−0.26, 0.60)	0.434

	Tubers & potatoes comp. scores^a,b,m^			Dairy products component scores^a,b,n^			Eggs & white meats comp. scores^a,b,o^			Vegetable oils component scores^a,b,p^		
	Mean (95% CI)	Diff (95% CI)	P-value	Mean (95% CI)	Diff (95% CI)	P-value	Mean (95% CI)	Diff (95% CI)	P-value	Mean (95% CI)	Diff (95% CI)	P-value
**Sociodemographic characteristics**												
Child gender												
Male	0.49 (0.33, 0.65)			4.03 (3.71, 4.35)			2.64 (2.32, 2.95)			5.59 (5.34, 5.85)		
Female	0.48 (0.33, 0.64)	−0.01 (−0.23, 0.22)	0.957	3.51 (3.21, 3.82)	−0.52 (−0.96,−0.07)	0.023*	3.01 (2.71, 3.32)	0.38 (−0.06, 0.81)	0.094	5.54 (5.29, 5.79)	−0.05 (−0.42, 0.30)	0.756
Child age												
3–4 years	0.49 (0.35, 0.62)			3.78 (3.52, 4.04)			2.83 (2.57, 3.09)			5.60 (5.39, 5.81)		
5–6 years	0.49 (0.27, 0.70)	0.00 (−0.25, 0.26)	0.978	3.72 (3.29, 4.15)	−0.06 (−0.56, 0.45)	0.827	2.84 (2.42, 3.26)	0.01 (−0.48, 0.51)	0.953	5.46 (5.12, 5.81)	−0.14 (−0.55, 0.27)	0.502
Maternal age												
< 25 years	0.61 (0.34, 0.89)			3.62 (3.08, 4.17)			2.87 (2.34, 3.41)			5.28 (4.84, 5.71)		
≥ 25 years	0.46 (0.34, 0.58)	−0.15 (−0.46, 0.15)	0.319	3.79 (3.55, 4.04)	−0.17 (−0.43, 0.77)	0.582	2.82 (2.58, 3.07)	−0.05 (−0.64, 0.54)	0.866	5.63 (5.43, 5.82)	0.35 (−0.13, 0.83)	0.157
Maternal level of education												
Incomplete SE	0.36 (0.09, 0.62)			4.18 (3.65, 4.70)			2.48 (1.96, 2.99)			6.03 (5.61, 6.45)		
Complete SE	0.51 (0.36, 0.66)	0.16 (−0.15, 0.46)	0.316	3.71 (3.41, 4.01)	−0.47 (−1.07, 0.14)	0.130	3.07 (2.77, 3.36)	0.59 (−0.00, 1.19)	0.051	5.50 (5.26, 5.74)	−0.53 (−1.02, −0.05)	0.032*
Complete TE	0.52 (0.30, 0.74)	0.17 (−0.18, 0.51)	0.352	3.59 (3.15, 4.02)	−0.59 (−1.28, 0.10)	0.094	2.58 (2.15, 3.01)	0.11 (−0.57, 0.79)	0.756	5.38 (5.03, 5.74)	−0.65 (−1.20, −0.09)	0.023*
**Anthropometric characteristics**												
Child weight status^c^												
Non overweight	0.45 (0.29, 0.60)			3.65 (3.34, 3.95)			2.84 (2.54, 3.14)			5.48 (5.23, 5.73)		
Overweight	0.49 (0.28, 0.70)	0.05 (−0.21, 0.31)	0.721	3.99 (3.57, 4.40)	0.34 (−0.18, 0.86)	0.198	2.65 (2.24, 3.06)	−0.19 (−0.70, 0.32)	0.462	5.70 (5.36, 6.04)	0.22 (−0.20, 0.64)	0.299
Obesity	0.60 (0.33, 0.86)	0.15 (−0.16, 0.46)	0.348	3.75 (3.22, 4.28)	0.10 (−0.52, 0.73)	0.740	3.10 (2.58, 3.63)	0.26 (−0.35, 0.87)	0.398	5.60 (5.17, 6.03)	0.12 (−0.38, 0.62)	0.630
Maternal weight status^d^												
Non overweight	0.58 (0.30, 0.86)			4.05 (3.49, 4.60)			2.72 (2.18, 3.27)			5.55 (5.11, 6.00)		
Overweight	0.48 (0.30, 0.66)	−0.11 (−0.43, 0.22)	0.523	3.73 (3.36, 4.09)	−0.32 (0.97, 0.33)	0.333	2.79 (2.44, 3.15)	0.07 (−0.56, 0.71)	0.829	5.43 (5.14, 5.72)	−0.12 (−0.65, 0.40)	0.645
Obesity	0.42 (0.19, 0.66)	−0.16 (−0.59, 0.27)	0.466	3.59 (3.11, 4.06)	−0.46 (−1.32, 0.39)	0.289	2.95 (2.49, 3.42)	0.23 (−0.61, 1.07)	0.589	5.71 (5.33, 6.09)	0.16 (−0.53, 0.85)	0.648
Maternal abdominal obesity^d^												
Absence	0.44 (0.22, 0.66)			3.49 (3.06, 3.92)			2.84 (2.42, 3.27)			5.65 (5.31, 6.00)		
Presence	0.52 (0.34, 0.71)	0.08 (−0.25, 0.42)	0.629	3.98 (3.61, 4.35)	0.49 (−0.18, 1.15)	0.152	2.82 (2.46, 3.19)	−0.02 (−0.67, 0.64)	0.957	5.49 (5.19, 5.79)	−0.16 (−0.70, 0.37)	0.549

	Palm oil component scores^a,b,q^			Red meats component scores^a,b,r^			Animal fats component scores^a,b,s^			Added sugars component scores^a,b,t^		
	Mean (95% CI)	Diff (95% CI)	P-value	Mean (95% CI)	Diff (95% CI)	P-value	Mean (95% CI)	Diff (95% CI)	P-value	Mean (95% CI)	Diff (95% CI)	P-value
**Sociodemographic characteristics**												
Child gender												
Male	4.96 (4.53, 5.38)			4.76 (4.30, 5.22)			5.66 (5.21, 6.10)			0.16 (0.07, 0.26)		
Female	4.40 (4.00, 4.81)	−0.56 (−1.15, 0.03)	0.065	5.09 (4.64, 5.53)	0.33 (−0.31, 0.97)	0.314	5.29 (4.86, 5.72)	−0.37 (−0.99, 0.25)	0.246	0.21 (0.13, 0.31)	0.05 (−0.08, 0.18)	0.439
Child age												
3–4 years	4.70 (4.36, 5.05)			4.90 (4.53, 5.28)			5.40 (5.03, 5.76)			0.19 (0.11, 0.27)		
5–6 years	4.58 (4.02, 5.15)	−0.12 (−0.79, 0.55)	0.735	4.99 (4.38, 5.61)	0.09 (−0.64, 0.82)	0.812	5.64 (5.04, 6.23)	0.24 (−0.47, 0.94)	0.511	0.19 (0.07, 0.32)	0.00 (−0.14, 0.16)	0.934
Maternal age												
< 25 years	4.03 (3.32, 4.75)			4.98 (4.20, 5.76)			5.15 (4.40, 5.91)			0.08 (−0.08, 0.24)		
≥ 25 years	4.80 (4.48, 5.13)	0.77 (0.02, 1.57)	0.056	4.92 (4.57, 5.27)	−0.06 (−0.92, 0.80)	0.892	5.53 (5.19, 5.87)	0.38 (−0.46, 1.22)	0.374	0.21 (0.14, 0.29)	0.13 (−0.04, 0.31)	0.135
Maternal level of education												
Incomplete SE	5.02 (4.33, 5.72)			4.88 (4.12, 5.64)			5.13 (4.39, 5.86)			0.24 (0.08, 0.39)		
Complete SE	4.69 (4.30, 5.09)	−0.33 (−1.13, 0.47)	0.417	4.88 (4.45, 5.31)	0.00 (−0.87, 0.87)	0.997	5.31 (4.89, 5.73)	0.18 (−0.66, 1.03)	0.668	0.19 (0.10, 0.28)	−0.05 (−0.22, 0.13)	0.607
Complete TE	4.39 (3.81, 4.96)	−0.63 (−1.55, 0.28)	0.172	5.07 (4.44, 5.70)	0.19 (−0.80, 1.19)	0.704	6.01 (5.40, 6.62)	0.88 (−0.08, 1.84)	0.074	0.15 (0.03, 0.28)	−0.08 (−0.29, 0.12)	0.417
**Anthropometric characteristics**												
Child weight status^c^												
Non overweight	4.64 (4.23, 5.04)			4.96 (4.52, 5.40)			5.60 (5.18, 6.03)			0.14 (0.05, 0.23)		
Overweight	4.46 (3.91, 5.01)	−0.18 (−0.86, 0.51)	0.616	4.70 (4.10, 5.30)	−0.26 (−1.01, 0.49)	0.499	5.36 (4.78, 5.94)	−0.24 (−0.97, 0.48)	0.510	0.20 (0.08, 0.32)	0.06 (−0.09, 0.22)	0.420
Obesity	5.10 (4.39, 5.80)	0.46 (−0.36, 1.28)	0.274	5.20 (4.43, 5.97)	0.24 (−0.65, 1.13)	0.600	5.21 (4.47, 5.96)	−0.39 (−1.26, 0.47)	0.374	0.33 (0.18, 0.49)	0.19 (0.01, 0.38)	0.037*
Maternal weight status^d^												
Non overweight	4.48 (3.74, 5.21)			4.73 (3.94, 5.53)			5.80 (5.03, 6.57)			0.33 (0.17, 0.50)		
Overweight	4.76 (4.28, 5.24)	0.29 (−0.57, 1.15)	0.510	4.99 (4.47, 5.52)	0.26 (−0.67, 1.20)	0.580	5.27 (4.77, 5.78)	−0.53 (−1.43, 0.38)	0.252	0.16 (0.06, 0.27)	−0.17 (−0.36, 0.02)	0.087
Obesity	4.72 (4.09, 5.34)	0.24 (−0.89, 1.37)	0.675	5.01 (4.33, 5.69)	0.28 (−0.96, 1.51)	0.661	5.41 (4.75, 6.07)	−0.39 (−1.59, 0.80)	0.517	0.11 (−0.02, 0.25)	−0.22 (−0.47, 0.04)	0.093
Maternal abdominal obesity^d^												
Absence	4.51 (3.94, 5.09)			5.08 (4.46, 5.71)			5.06 (4.46, 5.67)			0.19 (0.07, 0.32)		
Presence	4.79 (4.31, 5.29)	0.28 (−0.60, 1.16)	0.533	4.80 (4.27, 5.34)	−0.28 (−1.24, 0.68)	0.565	5.78 (5.27, 6.30)	0.72 (−0.21, 1.65)	0.129	0.19 (0.08, 0.30)	−0.00 (−0.20, 0.19)	0.967

Abbreviations: *PHDI-C* Planetary Health Diet Index for children and adolescents, *FECHiC* Food Environment Chilean Cohort, *DGV ratio* dark green vegetables ratio, *ReV ratio* red and orange vegetables ratio, *WC ratio* whole cereals ratio, *95% CI* 95% confidence interval, *diff* difference (i.e., regression coefficient), *SE* secondary education, *TE* tertiary education

^a^Estimates and p-values from linear regression models adjusting for all characteristics listed in the table plus dietary recall characteristics including: day of the dietary recall (weekday vs weekend/holiday), type of eating pattern (typical (i.e., recall from a regular day) vs atypical (i.e., recall from a special occasion such as celebrations, sickness or vacations)), and type of diet (normal (i.e., omnivorous diet with no dietary restriction of any kind) vs special diet (e.g., lactose free, gluten free, vegan or vegetarian diets)); n = 917

^b^Scores range from 0-10 points in all components, except in the DGV ratio and ReV ratio components, where scores range from 0-5 points

^c^Missing data for 1 child who refused to be measured

^d^Missing data for 40 participants; 38 mothers were pregnant and two refused to be measured

^e^Adjusted R-squared = −0.0015

^f^Adjusted R-squared = 0.0369

^g^Adjusted R-squared = 0.0187

^h^Adjusted R-squared = 0.0135

^i^Adjusted R-squared = 0.0087

^j^Adjusted R-squared = 0.0169

^k^Adjusted R-squared = 0.0071

^l^Adjusted R-squared = 0.0056

^m^Adjusted R-squared = −0.0104

^n^Adjusted R-squared = 0.0071

^o^Adjusted R-squared = 0.0032

^p^Adjusted R-squared = −0.0019

^q^Adjusted R-squared = 0.0143

^r^Adjusted R-squared = −0.0068

^s^Adjusted R-squared = 0.0077

^t^Adjusted R-squared = 0.0154

*p-value < 0.05

## Discussion

This sample of Chilean children had low PHDI-C total scores, suggesting low adherence to sustainable healthy diets. This was due to low consumption of *nuts & peanuts*, *legumes*, *vegetables, whole cereals* and *vegetable oils*; a lack of balance between *dark green* and *red & orange vegetables*, inadequate (either low or excess) consumption of *tubers & potatoes* and *eggs & white meats*, and excess consumption of *dairy products*, *palm oil*, *red meats*, and *added sugars*.

Our findings are consistent with those of Bäck et al. in their study of Finnish pre-schoolers (3–6 years, n = 862) [11]. They found that mean consumption of *nuts, legumes, vegetables, whole cereals*, and *vegetable oils* was lower than recommended, and consumption of *tubers & potatoes, dairy products, red meat,* and *added sugars* was higher than recommended by the EAT-Lancet Commission. Similarly, Gormaz et al. [7] reported that a representative sample of the Chilean population (≥ 2 years, n = 4920 of which 5% were children aged 2–5-year-old [31]) had low consumption of *nuts, legumes, whole cereals,* and *vegetable oils*, and high consumption of *dairy products, red meat,* and *added sugars*. Our findings also align with the results observed among European adolescents from the HELENA cohort study [32, 33] showing low adherence to the planetary health diet as measured by the PHDI (mean total score = 44.3 (95% CI 43.7, 44.9) points). Collectively, these studies reflect the nutrition transition away from minimally processed foods towards animal-sourced foods and ultra-processed products [34]. They suggest that to achieve sustainable healthy diets, strategies should target children’s low consumption of *nuts & peanuts, legumes, vegetables (particularly dark green vegetables),* and *whole cereals*, and high consumption of *dairy products, palm oil, red meats,* and *added sugars*. Implementation of triple-duty actions, such as school feeding programs based on sustainable healthy dietary guidelines [35] along with policies limiting access and exposure to unhealthy food and beverages [2], can help achieve this goal. In Chile, the introduction of the Food Labelling and Advertising Law at the end of June 2016 [36] may have reduced children’s intake of *added sugars* and *saturated fats* from ultra-processed products consumed at schools [37]; however, it is still unknown whether this policy has improved their adherence to sustainable healthy diets. Barriers to achieving this likely include the relatively low cost and increased availability of ultra-processed products [34] and significantly higher cost of healthy diets [38], particularly for low-medium income households [13] such as those in this study.

We found that children whose mothers were ≥ 25 years had significantly higher mean PHDI-C total score and higher mean *vegetables* component score compared to children with younger mothers. While the difference is small (3.4 points), it might be explained by a potentially lower socioeconomic status among mothers who bear children at a younger age [39] or by a potentially higher nutrition knowledge among older women compared to younger women [40]. Both variables have been associated with increased accessibility of fruits and vegetables and low home availability of unhealthy discretionary foods in a systematic review [13].

Additionally, we observed a negative association between the score for *fruits* and child age, which is consistent with a systematic review of determinants of children’s fruit and vegetable intake [41]. On the other hand, a significantly higher score for *fruits* was observed among children whose mothers had completed tertiary education compared to those whose mothers has not completed secondary education. This is in line with existing literature on predictors of children’s dietary intake that establish maternal education as a key determinant for home fruit accessibility and children’s nutrition knowledge [13]. Additionally, higher levels of education have been significantly associated with pro-environmental behaviours [42, 43]. We observed that the score for *vegetable oils* was significantly lower among children whose mothers had completed tertiary education. This may be explained by the negative association observed between maternal education and home availability of unhealthy snacks, which are usually high in vegetable oils [13]. We observed a significantly lower score for *dairy products* among females compared to males. This was explained by a slightly higher number of girls with *dairy products* consumption above the recommended intake (161 girls vs 131 boys). This is contrary to what has been observed in several developed countries where boys consume higher quantities of *dairy products* than girls [44]. Finally, a significantly higher score for *added sugars* was observed among children living with obesity. A possible explanation is lower consumption of added sugars among children living with obesity due to parents' efforts to restrict children’s dietary intake [45].

Previous studies have shown that maternal education is positively associated with home accessibility of vegetables and negatively associated with home availability of sugar-sweetened beverages [13]; however, probably due to a lack of socioeconomic heterogeneity in our sample, we did not observe a significant association between the score for *vegetables* or *added sugars* and maternal education.

### Strengths

A strength of this study is the use of the PHDI-C as it allowed us to describe children’s adherence to sustainable healthy diets in line with the EAT Lancet Commission recommendations [24], while taking into consideration their specific nutritional requirements [24]. The dietary data used in this study was collected by trained dietitians following the USDA five-step multi-pass method, which has been shown to reduce the risk of recall bias [20]. Additionally, the use of a photographic atlas of Chilean foods and culinary preparations [21] allowed the accurate estimation of portion sizes and the following estimation of children’s dietary intake. The level of detail obtained during data collection enabled researchers to link dietary data with all the information required to apply the PHDI-C as accurately as possible [24]. Finally, the large sample size and availability of child and maternal data enabled us to explore associations between PHDI-C scores and participants’ sociodemographic and anthropometric characteristics.

### Limitations

Our results come from a convenience sample of Chilean pre-schoolers living in low-medium income neighbourhoods of Santiago, Chile, and therefore, are not generalizable to children in other countries or from other parts of Chile. Another limitation is the use of single 24-h recalls for collecting dietary data, which are less likely to provide a measure of usual intake compared to multiple 24-h recalls [46], particularly for foods that are not consumed every day. Dietary recalls are also prone to recall bias [47]. We tried to reduce this risk by conducting the data collection process with trained dietitians and following the USDA multi-pass method [20], which led to 94.4% dietary recalls being classified as reliable. Finally, the absence of income data for participants in this study prevented us from exploring a direct association between PHDI-C scores and socioeconomic position and we used maternal education as a proxy [26].

## Conclusions

Adherence to sustainable healthy diets was low among this sample of Chilean pre-schoolers, particularly among those whose mothers were younger. These findings serve as a baseline for tracking changes in adherence to sustainable healthy diets over time and contribute to a growing body of literature calling for strategies to address children’s low consumption of plant-based minimally processed foods, and high consumption of animal-source foods and ultra-processed products. Further research is needed to assess whether Chile’s Food Labelling and Advertising Law has helped Chilean children transition towards healthier and environmentally sustainable dietary patterns.

## Supplementary Information

Below is the link to the electronic supplementary material.

Supplementary file1 (DOCX 26 KB)

## Data Availability

The de-identified data described in the manuscript, code book, and analytic code will not be made available publicly but can be made available on reasonable request. Proposals should be directed to the corresponding author, who will then pass the proposal on to members of the Center for Research in Food Environments and Prevention of Nutrition-related Chronic Diseases (CIAPEC)—INTA for deliberation and approval. To gain access, data requestors will need to sign a data access and collaboration agreement.
