# Supplementary material for: Chilean children’s adherence to sustainable healthy diets and its associations with sociodemographic and anthropometric factors: a cross-sectional study
Source: Eur J Nutr. 2024 Jun 3;63(7):2459–75. doi: 10.1007/s00394-024-03435-6 (PMC11490432; doi:10.1007/s00394-024-03435-6)
Supplement: Supplementary file 1 — Supplementary file1 (DOCX 26 KB) [file 394_2024_3435_MOESM1_ESM.docx]

# Supplementary material

**Article title:**

Chilean children’s adherence to sustainable healthy diets and its associations with sociodemographic and anthropometric factors: a cross-sectional study.

**Journal name:**

European Journal of Nutrition

**Authors’ names:**

Carolina Venegas Hargous,^1,2^ Liliana Orellana,^3^ Camila Corvalan,^4^ Claudia Strugnell,^1,5^ Steven Allender,^1^ Colin Bell.^1,2^

**Authors’ affiliations:**

1 Deakin University, Geelong, Australia, Global Centre for Preventive Health and Nutrition (GLOBE), Institute for Health Transformation.

2 Deakin University, Geelong, Australia, School of Medicine, Faculty of Health.

3 Deakin University, Geelong, Australia, Biostatistics Unit, Faculty of Health.

4 University of Chile, Santiago, Chile, Institute of Nutrition and Food Technology (INTA).

5 Deakin University, Geelong, Australia, Institute for Physical Activity and Nutrition (IPAN).

**Corresponding author:**

Colin Bell; Deakin University Waterfront Campus, Locked Bag 20000, Geelong, VIC 3220; [colin.bell@deakin.edu.au](mailto:colin.bell@deakin.edu.au)

# Supplemental figure 1. Participant flow chart.

Participants recruited at baseline

(n = 961)

Excluded (n = 3)

Participants with dietary data unavailable (n = 3)

Participants included

(n = 958)

Data available for analysis

- Dietary data: n = 958
- Sociodemographic data: n = 958
- Anthropometric data: n = 917
